# Interpersonal communication in healthcare

**Published:** 2018

**Authors:** CM Chichirez, VL Purcărea

**Affiliations:** * Carol Davila University of Medicine and Pharmacy, Bucharest, Romania

**Keywords:** skills, communication, marketing, health

## Abstract

Taking into account that the medical practice and health systems have evolved considerably nowadays, medical establishments require the implementation of marketing guidelines to help maximize performance, with beneficial effects from the social, economic, and medical point of view, to differentiate from competitors.

Communication is a fundamental clinical skill that, if performed competently and efficiently, facilitates the establishment of a relationship of trust between the medical staff and the patient-customer, a truly therapeutic alliance.

In addition to the medical personnel’s competence and the facilities at the doctor’s disposal, the willingness manifested during consultation, kindness, openness and attention are offered to patients-customers. The way medical personnel responds to their needs and requests is an element that boosts performance, contributing to an increase in the prestige of the medical unit and the growing interest of patients-customers in it.

Health services’ marketing includes activities carried out for the purpose of development, placement, pricing, or promotion of medical services. Communication is a very important component of the marketing mix. It is the instrument through which an entity participates in the exchange of information with the various components of the business environment, informs about their presence and the services offered, to create a favorable attitude and stimulate consumers to purchase services [**1**]. Organizations that provide health services, medical personnel (doctors and nurses) and auxiliaries (nurses, administrative staff, security guards) are a distinctive element of the marketing mix, adding value in healthcare through the way they interact with customers. Besides the training of medical staff and medical advancements in the field of diagnosis and treatment, the human component is a very important factor, over 40% of the therapy's success being provided by the patient-doctor relationship [**2**].

In health, the tools of the communicational mix are based on an interpersonal communication. Interpersonal (inter-human) communication was the first human spiritual tool of the socialization process [**3**] and is defined by Floyd [**4**] as being the communication that occurs between two people in the context of their relationship and that, as it evolves, helps to negotiate and define the relationship. In his work *“The psychology of communication. Theories and methods*”, Jean-Claude Abric [**5**] defines communication as “the ensemble of processes through which it carries out exchanges of information and of meanings between persons in a given social situation”. In the author’s opinion, “the communication cannot be conceived as a simple process of transmission, based on interaction, being always a transition between speakers: issuance and reception are simultaneous, the broadcaster being at the same time broadcaster and receiver and firstly broadcaster and receiver (the mutual is also valid)”. In his work *“Language as a means of social power*”, Blakar [**6**] emphasizes that “the broadcaster is a creator who, through his message, provides a whole set of information with respect to his own person, to his vision of the object of communication and the social situation he wishes and perceives, which will be perceived, interpreted and evaluated by the interlocutor, causing reactions, commitment and blocking them”. Certo Samuel [**7**] recommends the receiver “to always remain open in relation to people who communicate and be careful not to convey any negative attitude through their communication behavior” and, as receivers, they should try to take into consideration the value of the message that they receive, without taking into account attitudes toward the source. Otherwise, many valuable ideas will be lost if they allow messages they receive to be influenced by personal feelings.

It should be considered that medical system communication takes place in an environment that is complex, where favorable and adverse factors coexist and continuously exchange places and importance. In medicine, communication has many forms and can be seen in various situations, the most important of them undoubtedly being that between patient-doctor, which provides much of the data necessary for the establishment of the diagnosis. Interpersonal communication is born through the combination of verbal forms (an oral and written language), nonverbal (gestures, mimics, posture, movement, appearance) and paraverbal forms (by voice attributes accompanying the word, such as intonation, the inflection of voice, tone, rhythm, verbal flow). It can be influenced by a number of factors [**8**]: - the degree of closeness or spatial proximity; - the limits and the extent of physical contact in these relationships; - the friendly or authoritative style of communication; - the exchange of glances that form visual communication; - the volume and pace of the interactions; - the dynamics of reciprocal self-development.

Taking into consideration the importance of the information content of patient-doctor communication (the diagnostic process, treatment), the emphasis is more on verbal communication in the medical system. Non-verbal and paraverbal forms are important from the perspective of their emotional effect and reliable capital formation and sympathy that must exist between the two sides, these forms being devoid of semantic and logical values.

In their works, Cosman and Tudose emphasize the elements of specificity of communication in medicine. In the healthcare system, the relationship between the two parts, medical personnel-patient, is much more complex, involving a higher-level therapeutic communication of the existential type through the basal level of the therapeutic level. The higher-level therapeutic communication of the existential type is involved in medical communication because the medical act interferes with the patient's destiny, connected in turn by elements of uncertainty and individual instability [**9**].

On the other hand, the position of the two entities, medical personnel-patient respectively is different and unequal. This relationship is established between members of two distinct social groups in terms of their prestige, power, and guidelines. Thus, the doctor has an extremely high status, given the level of abstract information and specialized orientation towards the profession his full authority and monopoly being admitted. The social role of the patient legitimizes its temporary and permanent vulnerability, being forced to ask for support, assuming the doctor's inability to solve the health problem. In this situation, the patient is the most disadvantaged person, being under the influence of physical and mental suffering, feeling the disease as a source of uncertainty and insecurity, while the doctor is seen as a person with multiple qualities, full of energy and sometimes with magical powers. If, for the former, the disease is a scientific and objective problem, for the patient it is an emotional and subjective problem [**9**].

Thus, the doctor-patient relationship becomes a role, an asymmetric and consensual relationship, the doctor having a position of superiority, being the active element seeking a solution for the patient suffering from a disease, with the patient being the passive element that recognizes the authority of the doctor.

Depending on the degree of involvement of each part, Talcott Parsons [**10**] distinguishes between three situations in the doctor-patient relationship: - activity-passivity, in which the doctor is active and the patient is passive; - managing-cooperation, in which the patient follows the medical advice; - mutual participation, in which the doctor guides the patients through helping themselves.

George Ionescu [**11**] specifies that it is necessary to rebalance the doctor-patient relationship and to establish a communion through a mutual effort of understanding. Thus, the doctor will have to understand the subjective condition of the person in front of them and treat the patient regardless of their state, as an existence with a high degree of subjectivity. In turn, the patient will have to understand the meaning of the therapeutic act, to accept it with conviction and accept its efficiency and usefulness.

Under the given circumstances, the relationship between the two parts must, in time, become a special and true one, because the glue is a disease, which determines a particular behavior. The way each part will play their role can create the premises for a satisfactory and efficient relationship, or for a suspicious, frustrating and disappointing one [**12**]. Communication facilitates the establishment of a doctor-patient trust relationship, a real therapeutic alliance, with a purpose of improving the health status of the patient and the doctor's prestige, generally grown in the private and medical unit.

Another feature of the communication within the doctor-patient relationship is the fact that this relationship is direct, being carried out face to face, without the need of an intermediary and a meaningless formalism. The patient comes to the doctor with the hope that he will be understood and that his suffering will relieved, that the doctor will be competent and will deal with his personal health. Between the two, there is a continuous exchange of information, which will lead to the achievement of the objectives proposed and to finding out some answers on the state of the disease and its evaluation, the therapy proposed for its elimination and practical intervention procedures. This type of connection provides a real physical power over the medical team and the patient's psyche. [**13**].

Studies in the medical services domain noted that interactions between patients and healthcare professionals affect both patients' satisfaction and perceived quality of the medical services that they receive, and can contribute to a better performance of the medical unit [**2**] [**14**].

Communication in the medical act is an active process of transmission and reception of information, and, at least one of the partners of communication must have active listening skills, understanding of the message, and answering some questions for interpretation of non-verbal language, motivating the speaker to support the conversation [**15**].

In the medical domain, communication represents a fundamental clinical skill that involves the establishment of the therapeutic relationship, understanding the patient's perspective, exploring thoughts and emotions, and guiding them towards improving their health. The quality of the information obtained by the doctor during consultations is closely linked to the communication skills of the doctor and the patient. In literature, it is mentioned that the listening, explaining, and empathizing skills of the clinician can have a profound effect on the patient’s health status and functioning, as well as on their satisfaction regarding health care in the medical establishment [**16**] [**17**].

In the communication with the patient, listening and time (availability) are elements that must maintain attention to the speaker, regardless of the affective status, mode of cognitive operation. “Knowing to listen” is the first rule of the dialogue [**18**]. A number of rules have to be met for the listening to be efficient and profitable for the doctors. It needs to be active, total, empathic, receptive and with a certain criticism [**19**].

First, the listening must be active, which involves besides mental participation (attention and concentration) also a physical mobilization. For example, a too relaxed body posture makes memorizing and understanding difficult, while relatively uncomfortable positions generate a vigilant status propitious for good listening.

At the same time, listening must be total, meaning that in addition to receiving and understanding the verbal message, particular attention must be given to the non-verbal component (gesture signals). Thus, if the two partners of dialogue are positioned at the same level they will communicate better. In a dialogue, it is advisable to adopt an open, patient, and calm attitude amongst the conversation partners. Eye contact must be maintained, but not unnatural. Facial expression must be monitored so that the patient does not feel concerned, frustrated, or unmotivated. The listener must be empathic. Starting with self-awareness and continuing with other sightings, this cognitive and affective process allows the doctor to understand what the patient thinks and feels, to encourage them to express themselves openly and unrestrained. Empathy involves not just a mere sympathy or intuition of the patient's emotions, but identification with their feelings, with their biological and psychological status [**20**].

At the same time, the listening must be responsive (easy to pass over disturbing situations and treat the troublesome assertions of the speaker objectively) and involve some criticism (exaggerated tolerance towards the speaker will generate a form of dishonesty which will adversely affect the relationship).

In achieving effective communication, the medical staff must demonstrate availability towards patients - giving them some time to be scheduled and attended to according to the objectives and priorities, with the maximum performance. The availability displayed by the doctor during the consultation, the openness, attentiveness and helpfulness of the staff toward the patient and his family members are considered to be intrinsic attributes for any medical service supplying establishment and they must be maintained at such a high level of performance to differentiate them from other units [**21**].

The speaker expects a verbal message to provide a solution to an uncertainty or to confirm an expectation. This message must be accurate, fair, and appropriate to the situation of communication, informative, clear and prompt, respectful, without being unnecessarily formal, and without forgetting that the tone of voice matters [**19**]. Thus, any information offered to the patient removes a certain degree of uncertainty, and clarity is an example of a healthy way of thinking, as well as a proof of respect and a way to assume responsibility. Tangled and confusing answers affect the relationship. Located in front of the patient, the doctor aims to define the disease and to organize all the stages of the establishment of diagnosis and treatment. The oscillation, hesitation, or excessive delay in offering a solution has a negative influence on their relationship, the reaction being an essential condition of its effectiveness.

The establishment of the diagnosis and treatment, even though it remains the main purpose of the doctor-patient relationship, must be realized according to the patient’s need to be informed about items complementary to those of clinical importance, but from a medical perspective. If the patient is misinformed, they will be uncooperative, confused, dissatisfied, the context in which any medical act becomes stressful [**22**]. Communication with the patient must be suitable to their status, insight and possibilities associated with elements of support of a positive relationship. The acquirement of strong communication skills, necessary for the purpose of establishing real specialized therapeutic alliances, requires profound medical knowledge to diagnose and treat disease, the ability to gather information from the patient, interpersonal skills to respond to the feelings and concerns of the patient and the ability to create and maintain a therapeutic relationship as a concrete offer of information and medical education [**15**].

The professional competence of the doctor is demonstrated through in depth theoretical and practical knowledge and applicative ability in a creative activity of individualized, personalized, and human care.

The good philosophical training, psychological and pedagogical approaches require that the doctor be able to analyze the multitude of information and feelings, mindsets and reactions so that they can diagnose not only the health state but also the patient's typology level, their cultural and mental state. At the same time, they must reveal the ability to recognize subtle ways through which patients are trying to communicate their issues and concerns and actively investigate the ideas and their opinions on the health status. The doctor’s communication skills must continuously be learned and improved.

In the context of the communication relationship between the medical staff and the patient-customer, compliance with professional ethics is required, the two parts being owed to their status of collaborators and not a distinction between the vanquished and the victors. In the first half of the twentieth century, the Spanish professor B. Masci [**23**] developed the most elevated moral, medical document with the rules of medical ethics and deontology, which will always be respected and that sums up as it follows: 1. Honor your patient regardless of age. 2. Offer the same gratitude and attention to the poor as to the rich. 3. Respect your noble mission, beginning with your very own person. 4. Let your fatigue be enlightened by faith and love. 5. Never humiliate the sick, who is so humiliated by their illness. 6. Never forget that the secret entrusted to you about a disease is something holy that cannot be betrayed, offered to another person. 7. Do not see in your patients' worries a burden, a chore. 8. Never show incongruence at the success of the treatment on a sick person. 9. Not only benevolence but also science is required in the care of the sick. 10. Do not discuss medical prescriptions with the patient and never contradict them. You take away their confidence in medicine; you destroy their hope of healing.

The efficiency of communication is dependent on what type of relationship is established between the two partners, and the type of relationship, in turn, depends on the personality of each one of them [**5**]. The doctors determine their personality traits, having the freedom to choose their way of action so that the relationship with the patient is beneficial for both sides. In the healthcare system, communication becomes increasingly more of a therapeutic technique, a clinical skill that creates fundamental relationships and that can provide benefits to those involved, considerations for which the appropriation of high communicative skills must be a priority for health professionals.
